# From speech to thought: the neuronal basis of cognitive units in non-experimental, real-life communication investigated using ECoG

**DOI:** 10.3389/fnhum.2014.00383

**Published:** 2014-06-13

**Authors:** Johanna Derix, Olga Iljina, Johanna Weiske, Andreas Schulze-Bonhage, Ad Aertsen, Tonio Ball

**Affiliations:** ^1^Department of Neurosurgery, Epilepsy Center, University Medical Center FreiburgFreiburg, Germany; ^2^Department of Neurobiology and Biophysics, Faculty of Biology, University of FreiburgFreiburg, Germany; ^3^Bernstein Center Freiburg, University of FreiburgFreiburg, Germany; ^4^GRK 1624, University of FreiburgFreiburg, Germany; ^5^Department of German Linguistics, University of FreiburgFreiburg, Germany; ^6^Hermann Paul School of Linguistics, University of FreiburgFreiburg, Germany

**Keywords:** natural behavior, parietal cortex, prefrontal cortex, electrocorticography, high gamma mapping, autobiographical memory, idea unit, speech production

## Abstract

Exchange of thoughts by means of expressive speech is fundamental to human communication. However, the neuronal basis of real-life communication in general, and of verbal exchange of ideas in particular, has rarely been studied until now. Here, our aim was to establish an approach for exploring the neuronal processes related to cognitive “idea” units (IUs) in conditions of non-experimental speech production. We investigated whether such units corresponding to single, coherent chunks of speech with syntactically-defined borders, are useful to unravel the neuronal mechanisms underlying real-world human cognition. To this aim, we employed simultaneous electrocorticography (ECoG) and video recordings obtained in pre-neurosurgical diagnostics of epilepsy patients. We transcribed non-experimental, daily hospital conversations, identified IUs in transcriptions of the patients' speech, classified the obtained IUs according to a previously-proposed taxonomy focusing on memory content, and investigated the underlying neuronal activity. In each of our three subjects, we were able to collect a large number of IUs which could be assigned to different functional IU subclasses with a high inter-rater agreement. Robust IU-onset-related changes in spectral magnitude could be observed in high gamma frequencies (70–150 Hz) on the inferior lateral convexity and in the superior temporal cortex regardless of the IU content. A comparison of the topography of these responses with mouth motor and speech areas identified by electrocortical stimulation showed that IUs might be of use for extraoperative mapping of eloquent cortex (average sensitivity: 44.4%, average specificity: 91.1%). High gamma responses specific to memory-related IU subclasses were observed in the inferior parietal and prefrontal regions. IU-based analysis of ECoG recordings during non-experimental communication thus elicits topographically- and functionally-specific effects. We conclude that segmentation of spontaneous real-world speech in linguistically-motivated units is a promising strategy for elucidating the neuronal basis of mental processing during non-experimental communication.

## Introduction

Spontaneous language can reflect mental states and thus constitutes a fundamental link between externally-observable behavior and internal cognitive processes (Chafe, 1994, 2000, 2012). In the present study, we explored the utility of spoken language to investigate the neuronal correlates of higher-order cognitive functions. To this purpose, we analyzed real-world conversations from simultaneously-obtained video and intracranial electroencephalographic data.

Intracranial electroencephalography recorded for diagnostic purposes from the human brain includes both electrocorticography (ECoG) and stereo-electroencephalography and is now increasingly being used to study higher-order cognition. Such functions have been addressed as speech perception (Crone et al., 2001a; Canolty et al., 2007; Pasley et al., 2012) and production (Crone et al., 2001b; Towle et al., 2008; Bouchard et al., 2013), social interaction (Cristofori et al., 2012; Derix et al., 2012; Mesgarani and Chang, 2012; Caruana et al., 2013), and episodic (Burke et al., 2013) and autobiographical (Steinvorth et al., 2010) memory. Non-experimental ECoG approaches to study speech (Towle et al., 2008; Bauer et al., 2013; Ruescher et al., 2013) and social cognition (Derix et al., 2012) have lately been proposed, which allow studying brain activity of humans behaving in out-of-the-lab conditions. Recently, we presented a new approach to study real-life interaction between people based on ongoing simultaneous ECoG and monitoring video recordings obtained during pre-neurosurgical diagnostics of epilepsy (Derix et al., 2012). Such data encompass situations in which patients are engaged in naturalistic discourse and thus constitute a rich source of information about uninstructed, real-world social behavior. This allows conducting neurolinguistic studies based on concepts developed in psycholinguistic research on spontaneously-spoken language. ECoG is particularly well-suited for such investigations, as it combines a high temporal resolution with a high resistance against myographic artifacts (Ball et al., 2009; Derix et al., 2012).

For a start, we sought a way to break down long periods of continuous speech into comparable linguistic entities. Different approaches exist to split spoken language into meaningful constituents (Auer, 2010). For instance, segmentation into single words or phrase structures appears to be a direct and intuitive approach. Yet, if one aims to study such abstract phenomena as memory-related processing, longer units of about clausal length are most likely required (Dritschel, 1991). A speech unit of suitable length may be, e.g., the *prosodically*-defined “idea unit” (Chafe, 1980, 1985), later referred to as “intonation unit” (Chafe, 1994), which is identified in the course of speech by its cohesive intonation contour, or also the *syntactically*-defined “idea unit” (IU; Dritschel, 1991), identified as “a clause consisting of a finite verb plus all its modifiers.” We used the latter segmentation approach to extract cognitive units from ongoing speech in the present study. As previous literature indicates that the human capacity for short-term memory roughly corresponds to the length of an average syntactic clause (Pawley and Syder, 1983), we hypothesized that the message contained in an IU might be processed as a single entity, and that the underlying neuronal activity would reflect such cognitively-meaningful pieces of information.

Segmentation of speech into IUs as defined above or into comparable entities proved useful in psycholinguistic research on memory (Stafford and Daly, 1984; Stafford et al., 1987; Bangerter, 2000; Cuc et al., 2006; Muller and Hirst, 2010). However, only few IU-based studies exist in neurolinguistics (autobiographical narratives: Braun et al., 2001; effects of prior knowledge in memory processing: Maguire et al., 1999). All of them were conducted experimentally, and although a unit-driven approach is particularly well-suited to investigate spontaneous discourse (Dritschel, 1991), we are not aware of any IU-based neurolinguistic investigation under real-world conditions.

We here thus aimed to explore whether IU-segmented spontaneous, non-experimental speech as it occurs during conversations of ECoG-implanted patients in pre-neurosurgical evaluation is suited to investigate the underlying cognitive and neuronal processes. We transcribed several hours of conversations per patient, extracted IUs from the transcriptions, and assessed whether the obtained IUs could be classified into groups with clearly-defined functional differences, and whether such groups were comparable in terms of their basic features, such as the average temporal duration and word count. To elucidate functional differences between the IUs, we assigned them to subclasses based on the presence and type of memory content according to a previously-devised IU taxonomy by Dritschel (1991). These IU classes with different content were finally used to identify the underlying neuronal differences.

High-frequency oscillations of population activity are caused by delayed inhibitory feedback (Brunel and Hakim, 1999; Brunel, 2000) and shape the oscillatory properties of pairwise neuronal correlations (Helias et al., 2013). Neuronal activity in the high gamma range reflects spiking processes (Ray et al., 2008; Manning et al., 2009) and constitutes a direct and robust temporal, spatial, and functionally-specific index of event-related cortical activation (Crone et al., 1998; Ball et al., 2008; Cheyne et al., 2008). Previous intracranial EEG studies show that high gamma activity is a reliable marker for cognitive processing (Crone et al., 2011), such as in expressive and receptive speech (Crone et al., 2001a,b; Sinai et al., 2005; Perrone-Bertolotti et al., 2012) as well as during the involvement of memory functions (Jensen et al., 2007; Sederberg et al., 2007; van Vugt et al., 2010). We therefore focused our analyses on spectral magnitude modulations of ECoG recordings in this signal component.

## Materials and methods

### Subjects

Data were analyzed from three patients (two female: S1, S2 and one male: S3) who underwent temporary placement of intracranial electrodes for the purpose of pre-neurosurgical diagnostics of medically-intractable epilepsy. Electrodes were implanted for 1–3 weeks to localize the seizure onset zone and to evaluate the possibility of surgical treatment. The patients were video-monitored 24 h a day during this time period. All patients gave their informed consent that the recordings of neuronal activity and other data collected during the diagnostic procedure might be used for scientific purposes. The locations and numbers of implanted electrodes were determined with no concern of the present study and depended entirely on the individual clinical needs of the patients. All subjects had either left-hemispheric (S1 and S2) or bilateral (S3) speech dominance according to functional magnetic resonance imaging and electrocortical stimulation mapping (ESM). Subject details are summarized in Table 1.

**Table 1:** **Subject details**.

	**Age**	**Sex**	**Hand**.	**Lang. dominance**	**Implantation site of the 8 × 8 grid**	**Seizure onset zone**
S1	41	F	L	L	Left parieto-temporo-frontal	Left precentral
S2	49	F	R^*^	Bilateral	Left parieto-temporo-frontal	Left SMA
S3	57	M	A	L	Left fronto-parieto-temporal	Left parieto-occipital

Hand., handedness; Lang. dominance, language dominance; F, female; M, male; L, left; A, ambidextrous; R^*^, right-handed converted from left.

### ECoG recordings

The subdurally-implanted 8 × 8 platinum electrode grids had an inter-electrode distance of 10 mm and an electrode diameter of 4 mm. The grids of all three subjects covered parts of the left temporal, frontal, and parietal cortices (see Table 1, Figures 3A, 4A, 5A). Additional recordings were made in all subjects using subdural strip electrodes and/or depth electrodes. For comparability across subjects, we analyzed IU-category-related activity in grid recordings. Data from the subdural strips were inspected only for language mapping with high gamma activity (see below). ECoG was obtained using a clinical AC EEG-System (IT-Med, Germany) at a sampling rate of 1024 Hz. Data were high-pass-filtered with a cutoff frequency of 0.032 Hz, and low-pass-, anti-aliasing-filtered at 379 Hz. Synchronous monitoring audio and digital video recordings were acquired at a sampling frequency of 25 Hz and at a resolution of 640 × 480 pixels.

### ESM

ESM was performed using an INOMED NS 60 stimulator (INOMED, Germany). Pulse trains of 10 s consisting of pulses at 50 Hz and alternating-polarity square waves of 250 μs were applied systematically to pairs of electrodes. Bipolar stimulation was performed to identify non-overlapping pairs of electrodes with movement- and speech-related functions. The functionally-relevant contact(s) of the pair was (were) further identified using monopolar ESM. The intensity of the stimulus was gradually increased until a sensory, motor, or speech effect was induced. If no sensory (e.g., tactile), motor (stimulation-evoked movement or transient inability to move), or speech-related response (i.e., transient impairment of speech production and/or comprehension) could be observed at 15 mA (18 mA for speech functions), the stimulation was interrupted. Areas involved in receptive and expressive speech were localized using a battery of six tasks: counting, execution of body commands, naming everyday objects, reading, repetition of sentences, and Token Test. The subjects were unaware of the stimulation timing until the occurrence of the aforementioned functional effects. All stimulations were performed by the medical personnel at the University Medical Center Freiburg. See Ruescher et al. (2013) for more information.

### Acquisition and selection of IUs

In the ongoing digital video recordings, time periods were identified in which the patients were engaged in uninstructed, spontaneous conversations with at least one person. Dialog partners were visitors (friends, family members, or life partners) or medical staff (physicians and nurses). During the selected time periods, the patients were awake, alert, and they actively participated in conversations. The patients were neither eating nor extensively moving. The data were selected such that no ESM was performed immediately before or during the analyzed time periods, and no epileptic seizure occurred at least 30 min before and 30 min after these periods.

### Transcription

The audio signal was extracted from the digital audio-video mpg recordings of the selected conversation periods in wav-format using the Media Converter SA Edition 0.8. Orthographic transcriptions of the patients' speech were made by native speakers of German using PRAAT (Boersma and Weenink, 2014) whenever acoustic conditions allowed (i.e., when there were no strong background noises, no overlapping talk, and when the speech was loud and distinctive enough). The overall duration of the transcribed periods was 169.12 min for S1, 113.22 min for S2, and 36 min for S3, yielding 600 IUs in S1, 390 IUs in S2, and 141 IUs in S3 (Table 2).

**Table 2:** **Inter-rater reliability and the fractions of IUs in the different categories**.

		**IUs**	**MUs**	**nMUs**	**PMUs**	**nPMUs**	**AMUs**	**AFUs**	**Prosp. Mem**.	**Meta-mem**.	**Action**	**Eval**.	**Prop. Att**.	**Rep. Sp**.
S1	κ		0.53 ± 0.02	0.51 ± 0.02	0.54 ± 0.02	0.51 ± 0.02	0.22 ± 0.03	0.31 ± 0.03	0.38 ± 0.03	0.3 ± 0.03	0.56 ± 0.03	0.67 ± 0.03	0.31 ± 0.03	0.82 ± 0.03
	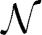 *o*	600	320	153	229	48	186	4	1	2	89	36	5	24
	%	100	53.3	25.5	71.6	15	81.2	1.7	0.4	0.9	47.8	19.4	2.7	12.9
S2	κ		0.59 ± 0.02	0.39 ± 0.02	0.41 ± 0.03	0.21 ± 0.03	0.38 ± 0.03	0.57 ± 0.03	N.A.	0.45 ± 0.03	0.69 ± 0.03	0.59 ± 0.03	0.54 ± 0.03	0.95 ± 0.03
	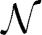 *o*	390	225	56	175	8	144	2	0	10	102	16	3	11
	%	100	57.7	14.4	77.8	3.6	82.3	1.1	0	5.7	70.8	11.1	2.1	7.6
S3	κ		0.71 ± 0.03	0.38 ± 0.03	0.62 ± 0.04	0.26 ± 0.04	0.47 ± 0.05	N.A.	0.66 ± 0.05	0.43 ± 0.05	0.85 ± 0.05	N.A.	0.89 ± 0.05	0.98 ± 0.05
	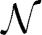 *o*	141	100	8	73	7	65	0	2	2	46	0	2	16
	%	100	70.9	5.7	73	7	89	0	2.7	2.7	70.8	0	3.1	24.6
All	κ		0.57 ± 0.01	0.48 ± 0.01	0.51 ± 0.02	0.4 ± 0.02	0.31 ± 0.02	0.37 ± 0.02	0.47 ± 0.02	0.42 ± 0.02	0.67 ± 0.02	0.64 ± 0.02	0.44 ± 0.02	0.9 ± 0.02
			Mod.	Mod.	Mod.	Fair	Fair	Fair	Mod.	Mod.	Subst.	Subst.	Mod.	Perf.
	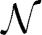 *o*	1131	645	217	477	63	395	6	3	14	237	52	10	51
	%		57	19.2	74	9.8	82.8	1.3	0.6	2.9	60	13.2	2.5	12.9

κ, Fleiss' kappa; 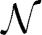
*o*, number of trials; %, share of the category in all IUs; N.A., no data from this IU category were available; MUs, memory units; PMUs, personal memory units; AMUs, autobiographical memory units; AFUs, autobiographical facts; Prosp. Mem., prospective memory units; Metamem., metamemory units; Eval., evaluations; Prop. Att., propositional attitudes; Rep. Sp., reported speech; Fair, fair inter-rater agreement (κ: 0.21–0.40); Mod., moderate agreement (κ: 0.41–0.60); Subst., substantial agreement (κ: 0.61–0.80); Perf., almost perfect agreement (κ: 0.81–1.00; Landis and Koch, 1977). All values were rounded to two digits after the decimal point.

### Classification of idea units (IUs)

**Figure 1:**
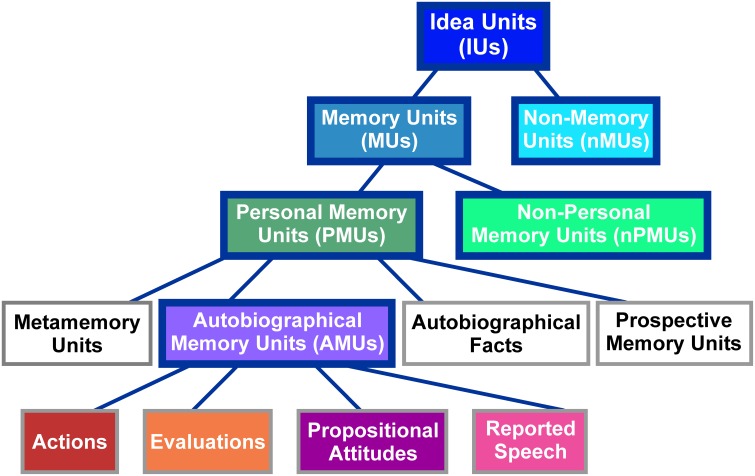
**Classification scheme of IUs employed in the present study**. Figure modified from Dritschel (1991). The abbreviations of category names are specified in brackets. The same colors as used here in the background of the individual panels are used in Figure 2 to depict the distribution of these categories in our spoken data. Categories employed for ECoG data analyses are highlighted by solid blue boxes.

IUs were identified in the transcriptions based on the definition of an IU as “a clause consisting of a finite verb plus all its modifiers” (Dritschel, 1991, p. 320). Thus, our identification of IUs relied on grammatical, and not on prosodic or semantic characteristics. IUs were classified into different categories according to the taxonomy proposed by Dritschel (1991). As illustrated in Figure 1, the classification at the highest level distinguishes between *memory units (MUs)*, which “implicitly or explicitly refer to the past,” (Dritschel, 1991, p. 320) and *non-memory units (nMUs)*. As Dritschel does not provide a definition of an nMU, we here defined this category as IUs which bear no explicit or implicit reference to the past. MUs were further subdivided into *personal memory units (PMUs)*, which, according to Dritschel (1991, p. 320) “implicate the self,” and *non-personal memory units (nPMUs)* “for the remaining memory units.” PMUs contain four subclasses: (1) *autobiographical fact units* with “some autobiographical/biographical information that need not be accessed in memory by event-related knowledge,” (2) *prospective memory units* expressing “a memory for satisfying some future plans,” (3) *metamemory units* relating “information about one's memory and the ability to access memories,” and (4) *autobiographical memory units (AMUs)* containing “an implicit or explicit self-reference to a past event or a collection of past events” (Dritschel, 1991, p. 321). The latter subclass was further subdivided into (i) *actions* “describing physical activity(ies) done or observed,” (ii) *evaluations* “expressing a previous interpretation of an action or feeling,” (iii) *propositional attitudes*, that “follow a verb which is expressed in the past tense and explicitly denotes a belief, thought, attitude, doubt or intention,” and (iv) *reported speech* “explicitly relating a statement made by a speaker in a previous conversation” (Dritschel, 1991, p. 326). See Supplementary Table 1 for examples of units in each of the described categories.

All IUs were assigned to the described categories by four independent raters. The raters were familiar with the taxonomy by Dritschel (1991) and evaluated whether an IU belonged to a category as either “positive,” “negative,” or “unclear.” While Dritschel (1991) used only positive and negative ratings, we introduced this latter option to account for IUs in which the context was either not sufficient to arrive at a clear decision, or for cases in which the IUs could not be assigned to any of the available categories. The “miscellaneous” category was only used for such cases at the level of PMUs and AMUs in the study by Dritschel. Since ambiguities could occur at all levels of classification, the rating “unclear” was allowed for all categories of IUs in the present study.

To assess the inter-rater reliability of the classification system, Fleiss' kappa (κ) (Fleiss, 1971) was calculated separately for each patient for the different IU classes. The degree of inter-rater reliability was evaluated based on the resulting κ-values, as proposed by Landis and Koch (1977).

An IU was considered as belonging to a category if it was assigned to the same category with an inter-rater agreement of at least 75% (i.e., either all four ratings were “positive,” or three were “positive” and one “unclear” or “negative”). This threshold was selected to restrict further analyses to the IUs for which the majority of raters agreed on the classification.

We detected on- and offsets of all transcribed IUs in the auditory signal and marked them manually in the neuronal recordings made simultaneously using the Deltamed Coherence PSG System (Paris, France). The median average durations, word counts, and their respective interquartile ranges (IQRs) were calculated in Matlab for the MUs, nMUs, PMUs, nPMUs, and AMUs. Statistical differences in the durations and word counts of the different IU classes were assessed using a non-parametrical Wilcoxon rank sum test (Gibbons, 2003), suited for unequal sample sizes (Sheskin, 2007).

### Data preprocessing, time-frequency analysis, and statistics

The recordings from the ECoG grids were re-referenced to a common average reference (CAR) across all grid electrodes. Whenever strip electrodes were analyzed, the recordings from these electrodes were re-referenced to a CAR across all strip electrodes in the respective lobe. For all channels and for each IU, we calculated event-related, time-resolved spectral magnitude values in a time frame between 4000 ms before and 3000 ms after IU onset to accommodate the entire duration of the IUs (Table 3) and to enable the analysis of memory retrieval processes which may begin well before the actual IU onset. Like in our previous ECoG studies (e.g., Pistohl et al., 2012; Ruescher et al., 2013), we employed a multi-taper method (Percival and Walden, 2010), using time windows of 500 ms, sliding windows of 50 ms, and 3 Slepian tapers. Trial-averaged, time-resolved spectral magnitudes were calculated in each subject for all available IUs together, as well as separately for IUs from the MU, nMU, PMU, nPMU, and AMU categories. Relative spectra were computed by dividing the time-resolved magnitude by the median baseline amplitude for each frequency bin. The baseline period was chosen between 4000 and 3000 ms before the onset of the respective IU category.

**Table 3:** **Overview of the length of the different IU categories**.

	**IU**	**MU**	**nMU**	**PMU**	**nPMU**	**AMU**
S1 duration (ms)	1174.8 ± 852.35	1268.55 ± 920.51	945.31 ± 761.2	1246.09 ± 916.94	1410.64 ± 1046.19	1250 ± 966.36
S1 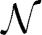 *o* words	5.6 ± 3.1	5.7 ± 2.9	5.8 ± 3.7	5.7 ± 2.9	5.9 ± 3.25	5.9 ± 3.1
S2 duration (ms)	1401.86 ± 848.7	1599.61 ± 949.44	1014.16 ± 419.82	1583.01 ± 934.44	2445.8 ± 1016.38	1633.3 ± 904.12
S2 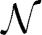 *o* words	5.4 ± 2.2	5.5 ± 2.3	4.9 ± 1.8	5.5 ± 2.3	6 ± 1.4	5.47 ± 2.3
S3 duration (ms)	1683.59 ± 1444.34	1839.84 ± 1513.89	1421.39 ± 787.37	1637.7 ± 1585.81	2216.8 ± 748.58	1637.7 ± 1603.55
S3 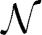 *o* words	6.4 ± 2.7	6.4 ± 2.8	5.8 ± 3.2	6.3 ± 2.8	7.3 ± 4	6.3 ± 2.9
All duration (ms)	1271.48 ± 970.71	1454.1 ± 1067.71	1003.91 ± 699.02	1387.7 ± 1067.38	1674.8 ± 1046.34	1433.59 ± 1087.9
All 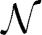 *o* words	5.6 ± 2.8	5.8 ± 2.7	5.6 ± 3.3	5.7 ± 2.7	6.1 ± 3.2	5.8 ± 2.8

Median durations and the numbers of words and their respective IQRs are shown for each subject (S1, S2, S3) separately and for all subjects together (“All duration” and “All 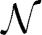
*o* words”). These average values were calculated as the median of the respective category in all subjects together. All values were rounded to two digits after the decimal point.

For statistical comparison between IUs from two different categories, the spectral magnitude in each trial was averaged over a time window of interest [corresponding to the period (i) 1 s before to IU onset or (ii) starting from IU onset to 1 s after IU onset] and averaged across the analyzed range of high gamma frequencies (70–150 Hz). We also performed the same analysis on theta (3–5 Hz) and alpha (8–12 Hz) frequencies to establish whether low-frequency effects parallel high gamma responses. The data were statistically tested in each of the analyzed time windows using a Wilcoxon rank sum test. The resulting *p*-values were false discovery rate (FDR)-corrected for multiple testing (Benjamini and Yekutieli, 2001) across the number of grid electrodes (64) and time windows (2) at a *q*-level of 0.05. In this way, we compared the categories MU vs. nMU, PMU vs. nPMU, PMU vs. nMU, and AMU vs. nMU.

We additionally performed a single-trial decoding analysis using a regularized linear discriminant analysis described in Pistohl et al. (2012) to assess whether PMUs and nMUs could be differentiated based on neuronal activity in single trials. The decoding was performed for each subject and recording channel, based on averaged high gamma magnitude values in the time windows (i) and (ii) separately. Like in our previous studies (e.g., Derix et al., 2012; Pistohl et al., 2012), the decoding accuracies were normalized to correct for bias due to unequal sample size of IU subclasses by averaging the class-specific decoding accuracies of the two classes.

To address the possibility of differential neuronal effects between the analyzed IU classes due to differences in syntactic complexity, we further correlated the time- and frequency-averaged high gamma activity in the same time windows as used for statistical comparison with the word count of the IUs, as word count has been shown to be a reliable measure for syntactic complexity (Szmrecsanyi, 2004). Resulting statistical values were FDR-corrected for multiple comparisons across electrodes at *q* < 0.05.

### High gamma mapping (HGM)

Since high gamma mapping has been suggested as a valuable adjunct to ESM to identify eloquent cortex in pre-neurosurgical diagnostics of epilepsy (Sinai et al., 2005; Leuthardt et al., 2007), we evaluated the topographic agreement of IU-related high gamma responses (below referred to as “high gamma mapping,” HGM) with the speech and mouth motor areas identified using ESM. The trial-averaged spectral magnitudes were calculated as described above. For statistical analysis, we averaged the data over the first 500 ms after IU onset and across the selected range of high gamma frequencies (70–150 Hz). Additionally, responses in theta (3–5 Hz) and alpha (8–12 Hz) frequencies were analyzed. We used a sign test, FDR-corrected at *q* < 0.001 for multiple testing across electrodes. The sensitivity and specificity of the HGM with IUs were calculated as in Ruescher et al. (2013). In addition to the 64 grid electrodes shown in Figures 3A, 5A for S1 and S2, respectively, one 1 × 6 and five 1 × 4-contact strips were analyzed in S1, and two 1 × 6 and four 1 × 4-contact strips in S2 (resulting in additional 26 and 28 in S1 and S2, respectively) for comparability with the HGM findings by Ruescher et al. (2013). The strip electrodes were implanted in frontal and interhemispheric areas. IU-related HGM results from these two subjects were compared to the sensitivity and specificity values from the data sets of non-experimental onsets of speech production analyzed in the same subjects by Ruescher et al. (2013). IUs in the present study and speech onset data in Ruescher et al. (2013) were extracted from only partially overlapping hours of conversation material, since the selection of the speech onset trials in this earlier study involved stricter inclusion criteria. For optimal comparability of the IUs with the data set reported in Ruescher et al. (2013), we re-analyzed this latter data set using the same parameters for spectral analysis as for the IUs.

### Anatomical assignment of ECoG electrodes and definition of brain areas

A T1-weighted magnetization-prepared rapid-acquisition gradient-echo (MPRAGE) image was obtained from each patient during the implantation period using a 1.5-T Vision magnetic resonance imaging (MRI) scanner (Siemens, Erlangen, Germany). After normalizing the MR images to a standard brain in MNI (Montreal Neurological Institute) space using SPM5 (Friston et al., 1994), electrode void artifacts, as well as the central, postcentral, and lateral sulci were identified and marked manually. The MNI coordinates of electrode positions were extracted and used for anatomical assignment to cortical areas based on a probabilistic atlas system (see Pistohl et al., 2012 and Ruescher et al., 2013 for details). The inferior parietal cortex (IPC) was defined using the Anatomy Toolbox version 1.6 (Eickhoff et al., 2005) and included the areas PF, PFm, PFt, PGa, PGp, and Fop (Caspers et al., 2006).

## Results

### Inter-rater reliability of IU classification

**Figure 2:**
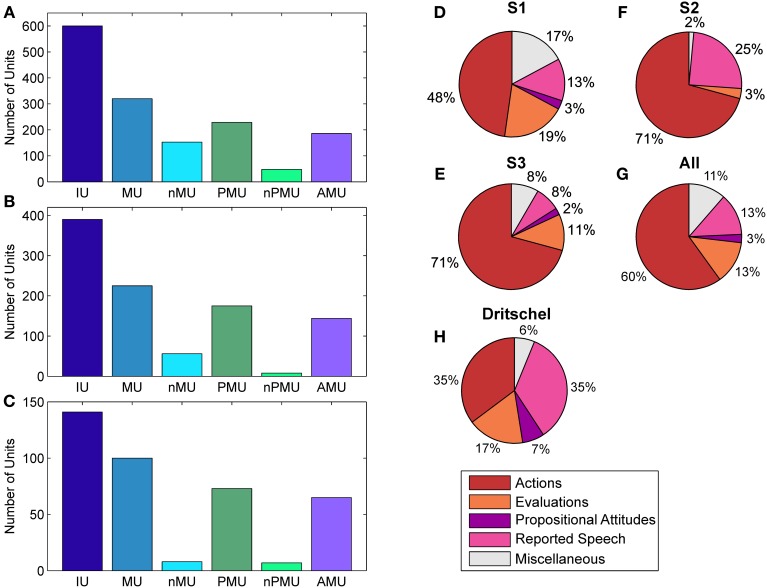
**Distributions of IUs across categories and subjects**. Panels **(A–C)** show distributions for categories which entered ECoG data analysis for S1–S3, respectively. Panels **(D–F)** show the fractions of “actions,” “evaluations,” “propositional attitudes,” and “reported speech” in the superordinate AMU category. **(G)** Summarizes the results in **(D–G)** as a proportion of summated IUs in the respective category of all subjects. **(H)** shows the same for the subclasses of AMUs in the study by Dritschel (1991), calculated as in **(G)** for comparison. The category “miscellaneous” in **(D–H)** contains AMUs not assigned to any other subcategory of AMU. Color coding as in Figure 1.

Fleiss' kappa κ was calculated for each IU category (see Figure 1), to evaluate the agreement of the raters' assignments (“positive,” “negative,” or “unclear”). Table 2 lists the κ-values for all subjects and categories. According to the interpretation of κ-values by Landis and Koch (1977), the average inter-rater agreement for MUs, nMUs, and PMUs was moderate (i.e., between 0.41 and 0.60), and the average inter-rater agreement for nPMUs and AMUs was fair (i.e., between 0.21 and 0.40). The κ-values for autobiographical fact units, prospective memory units, and metamemory units varied between moderate and fair inter-rater agreement. The subclasses of AMU reached higher agreement values than the other categories. The κ-values were substantial (i.e., between 0.61 and 0.80) for “action” AMUs and “evaluation” AMUs, and moderate for “propositional attitude” units. Assignment of “reported speech” AMUs elicited an almost perfect (κ = 0.9) inter-rater agreement. Further analyses were performed only with the IUs which elicited at least 75% of inter-rater agreement (see Methods). Figures 2A–C and Table 2 provide an overview of the numbers of IUs per category for all three subjects, as well as their relative portion in the total number of IUs per subject.

### Distribution of IUs over categories

The contribution of MUs to all IUs was 57% on average. It was similar in S1 and S2 (53.3% and 57.7%), while the percentage for S3 was higher (70.9%). The proportion of nMUs varied between 25.5% (S1), 14.4% (S2), and 5.7% (S3), and was 19.2% on average.

Also the fraction of PMUs and nPMUs in MUs was similar across subjects (average 74% and 9.8% for PMUs and nPMUs, respectively), ranging from 71.6% (S1) to 77.8% (S2) for PMUs, and from 3.6% (S2) to 15% (S1) for nPMUs. The distributions of MUs, nMUs, PMUs, and nPMUs could not be directly compared to the findings of Dritschel (1991), since this latter study did not report quantitative results for these categories. All IUs analyzed in the present study for each patient were extracted from several transcriptions and analyzed together. In the study by Dritschel (1991), however, each transcription was analyzed separately. We thus re-calculated the overall distribution of IU subcategories across all transcriptions in the data reported by Dritschel (1991) in the same way as we did for our transcriptions (cf. our Table 2 and Tables 2, 4 in Dritschel, 1991) for better comparability. AMUs formed the majority of PMUs in our data. The overall share of AMUs in the PMUs was 82.8% on average, and ranged between 81.2% in S1 and 89% in S3. The other categories of autobiographical fact units, prospective memory units, and metamemory units were sparsely present (on average 1.3%, 0.6%, and 2.9%, respectively). Overall, this proportion is consistent with the report by Dritschel.

The distribution of the four subclasses of AMUs (“action,” “evaluation,” “propositional attitude,” and “reported speech”) is shown in Figure 2 for each subject (Figures 2D–F), as well as for all subjects together (Figure 2G). The “action” category comprised the largest part of AMUs in all subjects, consistent with the earlier findings by Dritschel (Figure 2H), although the 35.5% of “action” AMUs observed by Dritschel (1991) is smaller than in our data (average 60%). S1 has conspicuously fewer “action” AMUs (47.8%) than S2 and S3 (both 70.8%). Dritschel found 35.2% of all AMUs to be “reported speech” units, clearly more than we observed in our transcriptions (12.9%). The frequency of these units in the present study also varied between subjects, S2 used them less often (7.6%) than S1 (12.9%) or S3 (24.6%). 13.2% of our AMUs were “evaluations,” slightly less than in Dritschel's data (17.3%). Only 2.5% of our AMUs were assigned to the “propositional attitude” category. This category was also underrepresented in Dritschel's transcriptions (6.7%). 11.4% of our AMUs remained unassigned, similar to the 6.2% of miscellaneous units in Dritschel's study.

### Number of words per IU and IU durations

Table 3 summarizes the average word count and durations for all analyzed IUs together and for the categories MU, nMU, PMU, nPMU, and AMU separately. The average number of words in one IU was 5.6 ± 2.8. This value was comparable across subjects: 5.6 ± 3.1 words in S1; 5.4 ± 2.2 words in S2, and 6.4 ± 2.7 words in S3. Word count was comparable for all subcategories of IU in all subjects (Wilcoxon rank sum test, *p* > 0.2).

The average IU duration was 1271.5 ms (IQR 970.7 ms), varying between 1174.8 ms (IQR 852.4 ms) in S1, 1401.9 ms (IQR 848.7 ms) in S2, and 1683.6 ms (IQR 1444.3 ms) in S3. Durations were comparable for some categories, e.g., for PMU vs. nPMU (*p* = 0.44 in S1, *p* = 0.63 in S2, and *p* = 0.11 in S3), while they differed for other categories such as nMU vs. MU (Wilcoxon rank sum test, *p* < 0.001 in all subjects).

### Functional topography of ECoG responses

**Figure 3:**
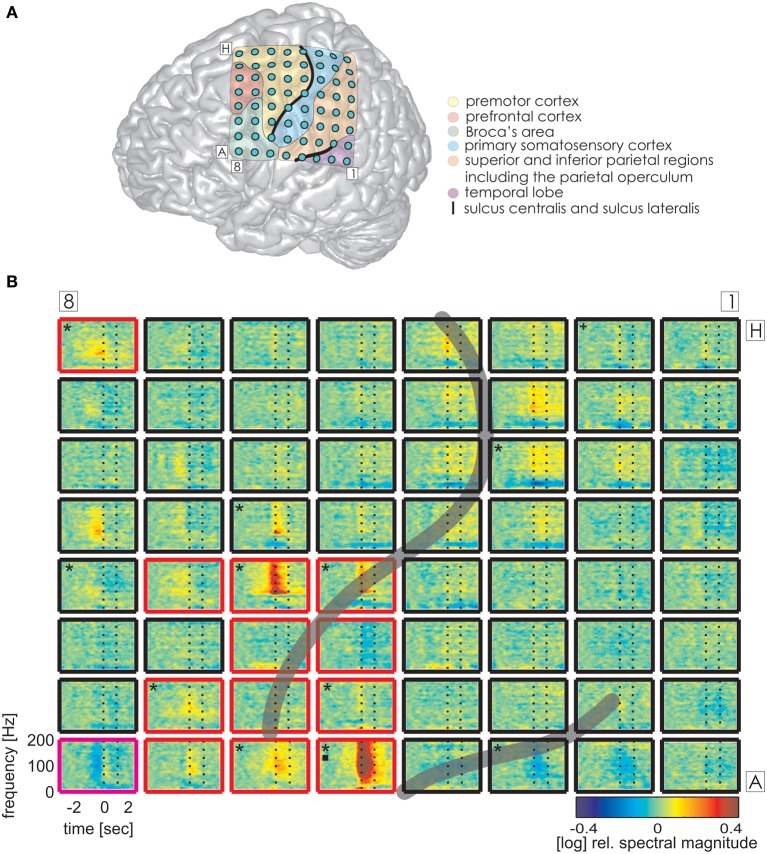
**Trial-averaged, time-resolved relative spectral magnitude changes of IU-related brain activity in S1. (A)** The individual location of the 8 × 8 electrode grid and the anatomical assignment of the electrodes (see Methods) are visualized on a Colin standard brain from SPM5 based on the MNI coordinates of the electrodes. Results of the anatomical assignment procedure are color-coded (see legend). **(B)** Cortical responses underlying the production of IUs averaged across 600 trials. Each of the 64 panels corresponds to the respective electrodes in **(A)**. The left vertical dashed line marks the onset (0 s), the right vertical dashed line marks the average end of the IU (1.174 s). Black asterisks mark electrodes with significant magnitude changes (Wilcoxon rank sum test, FDR-corrected at *q* < 0.001) in the high gamma (70–150 Hz), black squares in the theta (3–5 Hz), and black crosses in the alpha (8–12 Hz) frequency bands in the time period of 0–500 ms relative to IU onset. Potentially speech-related effects in ESM are indicated by the color of the electrode outline (red, mouth motor; magenta, cognitive speech impairment upon electrostimulation). The gray semi-transparent lines indicate the positions of the lateral and central sulci identified in the individual MRIs of the subjects.

**Figure 4:**
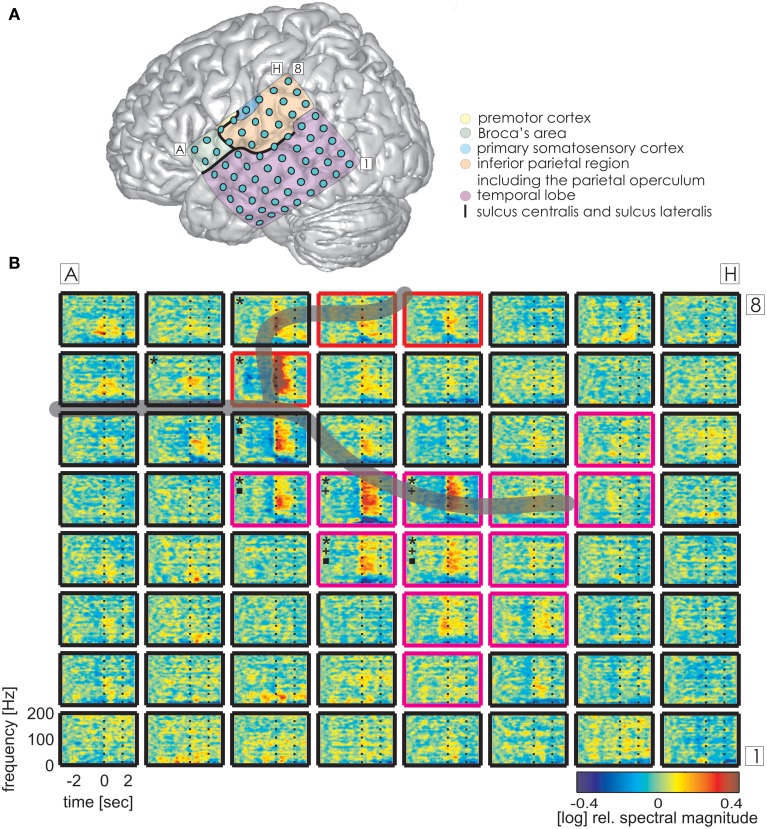
**Trial-averaged, time-resolved relative spectral magnitude changes of IU-related brain activity in S3. (A)** The individual location and anatomical assignment of the electrodes. **(B)** Cortical responses underlying the production of IUs averaged across 141 trials. Other details as in Figure 3.

**Figure 5:**
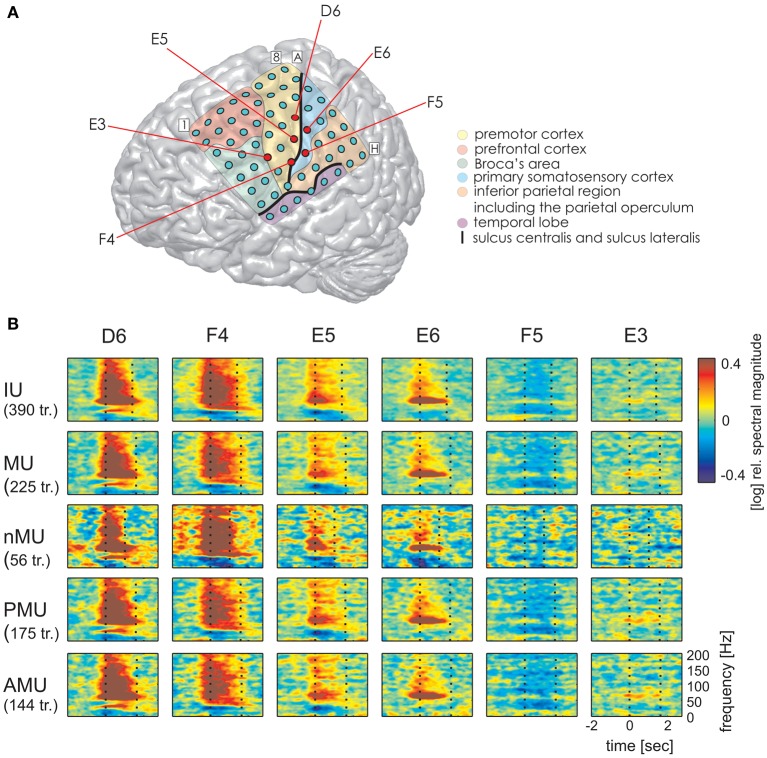
**Examples of typical time-resolved spectral magnitude changes underlying different IU categories at ESM-defined mouth motor electrodes (S2)**. Electrodes marked by red circles in **(A)** correspond to: electrodes with significant high gamma responses D6, F4, E3 (all IUs together) and electrodes without significant high gamma effects E5, E6, F5. Data are shown only for categories with at least 10 trials. Other details as in Figures 3, 4. Despite differences in durations of the IU classes (particularly between MUs and nMUs, see Table 3), speech-related responses are comparable across categories.

Time-resolved spectral analysis of the ECoG signals during the production of IUs revealed characteristic and significant changes in the high gamma frequency band, mostly increases but also decreases (spectra for S1, S3, and S2 are shown in Figures 3–5). As expected, significant IU-related effects were mostly observed in brain areas implicated in language and mouth motor functions, including Broca's area (BA 44 and 45) and the premotor cortex (cf. Figure 3A, 4A, 5A for exact anatomical locations). Spectral magnitude changes in different IU categories are shown in Figure 5 (S2) for electrodes with ESM-identified mouth motor functions localized by means of monopolar stimulation. Spectral response patterns were similar between categories, which likely reflects the predominance of common articulatory mechanisms involved in speech production regardless of the IU content.

The same analysis on theta and alpha frequencies elicited spatially sparser effects, some of which accompanied significantly increased high gamma activity in association areas including the IPC (increased theta activity at electrode A5 in S1, Figure 3), the superior temporal cortex (decreased theta activity at electrodes C5, C6, D4, E4 in S3, decreased alpha activity at electrodes D4, D5, E4 in S3; see Figure 4), and the lateral sulcus (decreased alpha activity at E5 in S3). Other effects in low frequencies which did not parallel those in high gamma frequencies took place in the superior middle and posterior temporal cortex (decreased theta activity at electrode H1 in S2, decreased alpha activity at electrodes H1–H5 in S2 (see Figure 5A for anatomical locations), and decreased alpha activity at electrode D4 in the superior middle temporal cortex of S3). There was decreased alpha activity at electrode H2 in the dorsal primary somatosensory cortex of S1 (see Figure 3). Decreased alpha activity could also be observed in S2 at electrodes F4 and G3 on the central sulcus and G4 in the IPC (an example of a spectral response at the electrode F4 is shown in Figure 5B, see Figure 5A for anatomical locations). Since low-frequency effects showed a looser spatial correspondence with ESM-identified speech and mouth motor areas (Figures 3, 4), we performed quantitative comparisons between ECoG and ESM effects selectively on high gamma frequencies.

### HGM and ESM

As overt speech production relies on both articulatory and cognitive functions, we considered electrodes with mouth-motor as well as cognitive speech-related effects in the comparison of ESM with HGM as potentially speech-relevant. HGM of IU trials revealed an average sensitivity of 44.4% and a specificity of 91.1%. Similarly, HGM performance with the speech onset data from Ruescher et al. (2013) reached a sensitivity of 43.3% and a specificity of 94.2%. All functional mapping results are summarized in Table 4. 5 electrodes per patient on average (5, 7, and 3 electrodes in S1–S3, respectively) showed significant high gamma responses which were not identified by ESM as responsible for speech or mouth movements.

**Table 4:** **Comparison of HGM of mouth motor and language functions for IUs and speech onset-related high gamma responses**.

	**Condition**	**Electrodes included in the analysis**	**Condition-related ESM responses**	***tp***	***tn***	***fp***	***fn***	**Sensitivity (%)**	**Specificity (%)**
S1	IU, 600 trials	73	15	8	53	5	7	53.3	91.4
	Speech, 50 trials	73	15	8	58	0	7	53.3	100
S2	IU, 390 trials	75	17	6	53	7	9	40	88.3
	Speech, 110 trials	75	17	5	53	7	10	33.3	88.3
S3	IU, 141 trials	64	15	6	46	3	9	40	93.5
Mean	IU, all trials	70.7	15.7	6.7	50.7	5	8.3	44.4	91.1
	Speech	74	16	6.5	55.5	3.5	8.5	43.3	94.2

tp, number of true positive electrodes; tn, number of true negative electrodes; fp, number of false positive electrodes; fn, number of false negative electrodes (see Ruescher et al., 2013 for more information). Statistics for IU- and speech-onset-related responses within the first 500 ms after onset are reported. The high sensitivity and specificity of HGM for speech production indicate that the present settings for ECoG data analysis are well suited for speech mapping under non-experimental conditions. The relatively good sensitivity and specificity of the IU-based approach shows that it is also suitable for identification of cortical areas supporting speech-related functions. The higher number of false positive electrodes in the IU data set than in the speech onset data suggests that additional processes may be involved in the production of IUs.

### Category-specific effects

**Figure 6:**
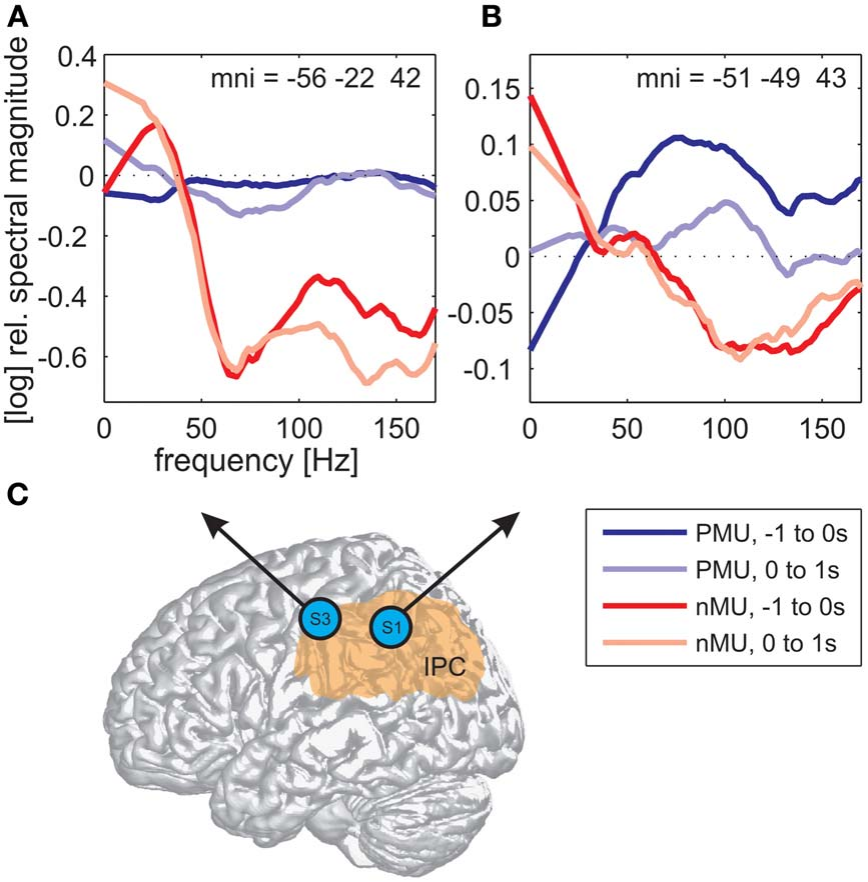
**Differences in high gamma magnitude underlying the production of PMUs vs. nMUs in the inferior parietal cortical (IPC) region of S1 (electrode E1) and S3 (electrode F8). (A,B)** Show stronger high gamma (70–150 Hz) magnitude in PMU than in nMU trials. Magnitude differences in subjects S3 **(A)** and S1 **(B)** were significant at one electrode in the IPC region of each subject (Wilcoxon rank sum test, FDR-corrected at *q* < 0.01) before IU onset (−1 to 0 s; dark red traces for nMUs and dark blue traces for PMUs), and also during the production of the IUs (in the first second after IU onset; light red traces for nMUs and light blue traces for PMUs). Data were smoothed using a first-order Savitzky-Golay filter with a bandwidth of 42 Hz. Electrode positions are visualized on a standard brain from SPM5 based on their MNI coordinates in **(C)**, the approximate extent of the IPC is indicated in orange.

Alongside the category-independent responses described above, differences in high gamma activity were observed in the IPC region and in the prefrontal cortex (PFC) between IU categories with vs. without memory content. The magnitude of high gamma activity in the IPC region was consistently smaller in nMU trials than in MU/PMU/AMU trials both in the 1-s time interval before and after IU onset (Figure 6). These parietal effects were significant (see Methods) in electrodes E1 from S1 (MNI: -51/-49/43, probability for IPC (PFm): 50%, probability for IPC (PGa): 30%, probability for IPC (PF): 30%, cf. Figure 3A for electrode position) and F8 from S3 (MNI: -56/-22/42, probability for Brodmann area 2: 60%, probability for IPC (PFt): 60%, probability for Brodmann area 1: 50%, cf. Figure 4A for electrode position). There was a similar IPC response pattern in electrode H7 from S2 (see Figure 5A for electrode position), yet the cross-category differences in this subject were not significant. Out of all subjects and time windows, the identified difference between PMUs and nMUs could only be decoded in S1 and only with a relatively low accuracy of 61.6% during the second before IU onset (*p* = 0.0029, Bonferroni-corrected for the number of grid electrodes and the number of time windows).

**Figure 7:**
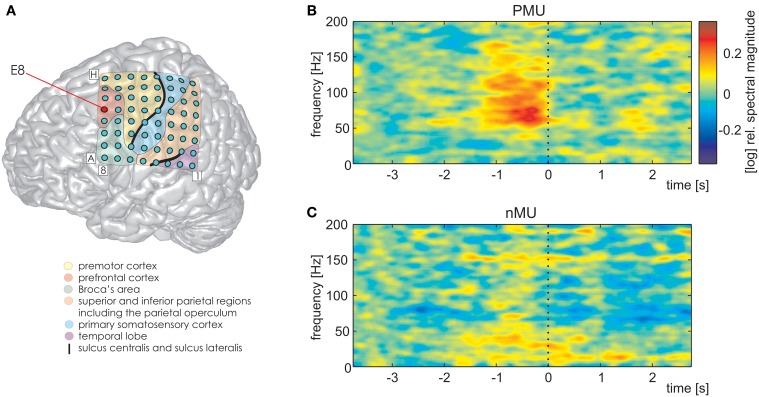
**Differential PMU- and nMU-related responses in the left prefrontal cortex of S1**. Red circle in **(A)** marks the prefrontal electrode with significant differences (Wilcoxon rank sum test, FDR-corrected at *q* < 0.01) between the two categories. **(B)** PMU-related responses, **(C)** nMU-related responses. High gamma spectral magnitude was stronger in IUs with personal memory content than in those without such content. Other details as in Figures 3–5.

We further detected one PFC electrode in S1 (Figure 7A, MNI: −36/20/50, no probabilistic assignment available, cf. electrode E8 in Figure 3A) with a significantly larger magnitude of high gamma activity within a second prior to the onset of IUs with memory content (MUs and PMUs, Figure 7B shows PMU-related effects) than without memory content (nMUs, Figure 7C). A similar significant effect could be observed in S2 at electrode A1 in the dorsomedial PFC. It consisted of significantly stronger high gamma activity during the first second after IU onset in the PMU vs. nMU and in the MU vs. nMU contrasts. There were also reproducible effects in alpha frequencies in the PMU vs. nMU comparison in the time window of 0–1 s relative to IU onset in both subjects. As opposed to the aforementioned gamma effects, the level of alpha activity was significantly lower in PMUs than nMUs at electrode D8 in S1 and at electrode A1 in S2.

Additionally, there were category-specific differences in high gamma activity in the anterior/middle (S3) and posterior (S1) superior temporal cortex in the time period of 0–1 s relative to the onset of IU production. Electrode B1 in S1 showed a significantly higher level of gamma activity in PMUs than in nPMUs, and electrodes A1 and B6 in S3 showed a contrary response with less gamma activity in the PMU than in the nPMU data. Electrode A1 in S3 had shown less gamma activity during conversations with the life partner than with the physician in our previously-published study (S3 in Derix et al., 2012). Thus, modulations of gamma activity at this electrode may reflect self-referential processing.

High gamma magnitude at the IPC and PFC electrodes with the aforementioned memory-related effects showed no significant correlation (Spearman's correlation, *p*-values FDR-corrected at *q* < 0.05) with the number of words in the IUs. Therefore, an explanation of these differential responses by systematic differences in syntactic complexity defined as the number of words (Szmrecsanyi, 2004) is unlikely.

Reproducible effects could be observed in the temporal cortex in the theta frequency range. These consisted of stronger activation in PMU than in the nPMU data in the posterior superior (electrode B1 in S1, see Figure 3A for electrode location), middle superior (electrode B6 in S3), and anterior inferior (electrode A1 in S3) temporal cortex (see Figure 4A for electrode locations). Furthermore, significant differences in theta frequencies occurred between MU vs. nMU, PMU vs. nMU, and AMU vs. nMU categories in the anterior inferior temporal cortex of S3, who had been investigated in our earlier study (Derix et al., 2012). The increased levels of theta activity in the memory-specific IU conditions at electrode C2 in this subject are consistent with our previously-expressed hypothesis that theta responses in the anterior temporal lobe may reflect autobiographical mnemonic processing (Derix et al., 2012).

All described effects were found outside the epileptic seizure onset zone and outside areas with language and mouth motor functions defined by the ESM and HGM procedures.

## Discussion

Implementation of study paradigms which are relevant to and representative of real-world situations is of central importance to understanding natural human cognition (Kingstone et al., 2003; Zaki and Ochsner, 2009; Maguire, 2012; Przyrembel et al., 2012; Stanley and Adolphs, 2013). To be able to capture neuronal processes which are grounded in real-life experiences, researchers more and more frequently employ such stimuli as longer and increasingly naturalistic text passages, Hollywood movies and video recordings of interacting individuals, or they place subjects in real-life-like environments such as highly detailed virtual simulations of face-to-face communication or traffic situations (see Spiers and Maguire, 2007; Mar, 2011; Borghini et al., 2012; Konvalinka and Roepstorff, 2012; Maguire, 2012; Schilbach et al., 2013 for reviews). Here, we explored the concept of “idea units” (IUs) as a way to get a handle on differential cognitive functions involved in non-experimental, real-life speech production. To this end, we transcribed continuous speech of ECoG-implanted patients, subdivided these data into syntactically-meaningful chunks of information (IUs), classified the obtained IUs according to their mnemonic content, and analyzed the underlying neuronal activity.

### Applicability of the investigated IU concept to simultaneous ECoG/video data

Spontaneous ECoG data are obtained for pre-neurosurgical diagnostics during everyday hospital life. While implanted with electrodes, patients are confined to bed for safety reasons and have to stay under constant video and audio surveillance by medical personnel. This can be expected to influence the patients' behavior and topics of conversation. Thus, we refrain from calling these unusual life circumstances “natural” but rather employ the terms “real-life” or “real-world.” The total recording time is limited to the time period of invasive monitoring (1–3 weeks), yet the recorded social situations are diverse. It was our aim to establish how many IUs can be collected from such data, whether they can be subdivided into functional subclasses, and how these speech data compare to IUs produced by previously-reported subjects in non-clinical settings. Our results showed that the patients' everyday dialogs contained sufficient amounts of IUs for elaborate behavioral and neurophysiological analyses.

The IU approach applied in the present study (Dritschel, 1991) allowed classifying IUs according to the different types of mnemonic content with a fair to perfect inter-rater agreement (Table 2). There was very good agreement for the AMU subclasses “action,” “evaluation,” and “reported speech.” Only fair agreement could be achieved for the categories nPMU, AMU, autobiographical fact units, and the AMU subclass “propositional attitude.” We employed a threshold of 75% inter-rater agreement to define functional categories for consecutive analyses. With this inclusion criterion, we were still able to obtain large numbers of trials in the major IU subclasses (Figures 2A–C), including MU, nMU, PMU, AMU, and “action” AMU. Thus, on the one hand, Dritschel's method could successfully be applied to our data. On the other hand, improvements are desirable in the reliability of ratings and in the level of detail of IU classification, for which further refined taxonomies (Bangerter, 2000; Cuc et al., 2006) and alternative segmentation methods (see Outlook) may be useful.

As to the distribution of IUs across the different subclasses, most IUs had mnemonic content (assigned to MU). The majority of them contained an explicit or an implicit reference to self (assigned to PMU), and most PMUs referred to a past experience (AMU). Most of those contained references to past actions (“action” AMU). Other PMU categories (metamemory units, autobiographical fact units, and prospective memory units) were covered only sparsely. Overall, our results are in keeping with those reported by Dritschel (1991) in healthy subjects during different real-life conversational situations. The somewhat larger share of “action” AMUs in our data than in the study by Dritschel (cf. Table 4 in Dritschel and our Table 2, Figures 2D–H) may reflect differences in the individual manner or contents of conversations between subjects, and/or it may be attributable to our more strict inclusion criteria.

The average number of words in an IU roughly corresponds to previous observations (Chafe, 1994). The number of words in our study was comparable across different IU subclasses, and the average durations were comparable across different MU subclasses (Table 3). Interestingly, the durations of MU subclasses were around 200 ms longer than those of nMUs (Wilcoxon rank sum test, *p* < 0.001). An explanation for this may be that speech with mnemonic content is slower due to memory retrieval processes. It may be interesting to address this putative difference in future psycho- and neurolinguistic studies.

The employed taxonomy allows classifying IUs according to several types of memories. Still, its major limitation is that it does not subdivide nMUs and nPMUs into further functional subclasses, which could provide useful counterparts to the different types of IUs with mnemonic and self-referential content. Theoretical research on subclassification of these kinds of IUs is hence desired. A further observation which may be relevant for future research is that, while there were many trials in the nMU and MU categories and in the two major (sub-)fractions of MUs (PMU and AMU), some IU classes are underrepresented in spontaneous communication. Since we obtained only few IUs from the available data and in the autobiographical fact units, in prospective memory units, in the different subclasses of AMUs (cf. Figure 2 and section “Distribution of IUs over categories”), we did not perform further quantitative analyses on these types of IUs. Considerably more extensive amounts of spoken data will be required to elucidate the neuronal correlates of these IU classes during real-world communication.

As is illustrated in Figure 1, the taxonomy by Dritschel (1991) classifies IUs based on a single hierarchy. However, as one of the reviewers has pointed out, a fine-grained description of semantic differences between various kinds of units will most likely comprise multiple dimensions. Development of theoretical approaches to IU classification and tests of their biological validity will be a valuable endeavor to which various lines of research can contribute.

### IU-related brain responses

Our aim in the second part of the study was to elucidate the neuronal activity underlying IUs as defined above. In all subjects, we observed prominent neuronal activations related to the production of IUs in such speech-related brain regions as Broca's area, the superior temporal gyrus, and the premotor cortex (Figures 3–5). The topography of the observed effects was consistent with research in healthy subjects (Pulvermüller and Fadiga, 2010; Price, 2012). Like in previous experimental ECoG studies on speech production, there were significant increases in the high gamma band (Crone et al., 2001b; Towle et al., 2008), often accompanied by decreased onset-related activity in alpha frequencies (Wu et al., 2010; Toyoda et al., 2014), although low-frequency effects seldom reached significance in our analysis. High gamma responses in the majority of electrodes were most pronounced around the onset of IUs and persisted over the entire average duration of the IUs. The sharp and accentuated change of activity around IU onset was striking, considering that IU onsets did not necessarily coincide with the start of speech production. This might be an indication that IU boundaries indeed have clear representations in brain activity. Since we did not account for the temporal distance of IU onset to the start of the respective speech production epoch (e.g., in the sense defined in Ruescher et al., 2013), future research will be required to disentangle the contribution of articulatory onset-related effects from those specific to the onset of syntactic constructions. Importantly, IU-related neuronal responses were equally well-visible in all IU categories for which the amount of the gathered data permitted trial-averaged spectral analysis (Figures 2A–C). The similarity of responses across the different IU categories in ESM-identified mouth motor areas (Figure 5B) indicates articulation-related processes common to the production of all classes of IUs. Taken together, these findings provide initial evidence that IUs are useful and appropriate basic elements for investigating the neuronal correlates of speech production under real-world conditions.

### Suitability of the present approach for language mapping

To establish whether the present IU-based approach is suited to identify cortical areas which support expressive language functions, we compared the topography of IU-related high gamma responses with the results of ESM, as well as with a data set of non-experimental speech onsets previously obtained for HGM of expressive speech (Ruescher et al., 2013). We found that IU-related responses had a high specificity (91.1%) and a moderate sensitivity (44.4%) for speech/mouth motor areas identified using electrocortical stimulation (Table 4). Thus, the present IU-based approach may provide a promising starting point for the development of adjuncts to experimental as well as other non-experimental approaches to define eloquent language cortex in pre-neurosurgical diagnostics (Ojemann and Whitaker, 1978; Sinai et al., 2005; Ruescher et al., 2013). Importantly, IU-related neuronal effects were not only observed in the classical speech areas but also in association areas including the PFC and the IPC regions. This suggests that there may be additional higher-order processes during IU production which may remain undetected by ESM. Further investigation is needed to address this issue.

The sensitivity and specificity of the proposed IU-based method was comparable to the HGM results obtained with a previously-published data set of speech onsets (Ruescher et al., 2013) in our S1 and S2 (P2 and P1 in Ruescher et al., 2013, respectively). Our re-analysis of the speech onset data using the same parameters as for IUs revealed a specificity of 94.2% and a sensitivity of 43.3% (Table 4). Interestingly, the present results for both data sets exhibited a higher sensitivity for ESM-defined speech areas than in the earlier report by Ruescher and colleagues. Note that the latter study aimed to develop a common mapping approach which would readily be applicable for mapping upper- and lower-extremity motor and language functions in a clinical environment. In the present study, however, we focused on optimizing the parameters of neuronal data analysis specifically for the purpose of language mapping. Exploration of other ECoG signal components, time windows, and alternative further parameters for neuronal data analysis may be of interest, as can be seen from the comparison of our speech mapping results (Table 4) with the effects observed by Ruescher et al. (2013). Together with their findings, our results suggest that optimal identification of speech and extremity motor functions may have different requirements for the analysis of ECoG data. This observation may be of consequence in achieving maximally-precise definitions of eloquent cortex in pre-neurosurgical diagnostics using HGM.

### Category-specific IU-related brain responses in the IPC

We observed differential modulations of activity in the parietal cortex depending on the presence or absence of memory content in the IUs. A comparison of the trial-averaged spectral magnitude in high gamma frequencies revealed consistent differences between nMU and MU/PMU/AMU trials in the IPC region, as is shown in Figure 6 on the example of nMUs and PMUs. These differences occurred both before and after IU onset, they were significant in S1 and S3, and started prior to gamma activation measured in articulation-related areas.

The observed memory-related activity in the IPC region agrees well with results from previous studies pointing to an important role of the parietal cortex in mnemonic processing (Wagner et al., 2005; Vilberg and Rugg, 2008). According to Svoboda et al. (2006), the lateral parietal cortex and the temporo-parietal junction form integral parts of the autobiographical memory network. Notably, we found IU-related reduced high gamma activity in the IPC region in the non-memory condition, compared to a steadily higher level of activation in the memory trials (Figure 6). One would expect such a difference if the IPC supported ongoing memory-related processing which was briefly interrupted by the occurrence of non-memory content.

Vilberg and Rugg (2008) proposed that the parietal cortex may serve as an “episodic buffer” (Baddeley, 2000) responsible for binding information from sensorimotor systems and from long-term memory into a temporary episodic recollection. Following this notion, it seems plausible that the buffer “empties” during the processing of non-memory content, which may explain the reduced gamma-band responses related to the nMUs in our study. Future investigation is needed to address this putative memory-retrieving mechanism.

### Category-specific IU-related brain responses in the PFC

A handful of fMRI studies which have been conducted to identify the neuronal correlates of thoughts show that the PFC is sensitive to their content. For instance, Spiers and Maguire (2006a,b) proposed and evaluated an approach to study cognitive units by correlating the content of the subjects' utterances in *post-hoc* oral reports about their experiences during navigation in a virtual-reality environment with the neuronal activity recorded during these experiences. Among other effects, these authors reported higher levels of activation in the medial PFC related to route planning (Spiers and Maguire, 2006b) and during Theory-of-Mind (ToM) recollections (Spiers and Maguire, 2006a) compared with several other categories. In a different fMRI study from the same group, Bonnici et al. (2012) asked the subjects to recall rich and vivid memories of recent (2 weeks ago) or remote (10 years ago) events, and found that the latter were more readily detectable in the ventromedial PFC than the former ones.

In the present study, we observed significantly higher levels of high gamma activity for memory- vs. non-memory-related IUs in the PFC of both subjects in whom this cortical region had electrode coverage (S1 ans S2; Figure 7 shows an example of such differential PFC responses for S1 in the PMU vs. nMU contrast). These effects are in line with previous neuroimaging literature pointing to the contribution of the PFC to mnemonic (Maguire et al., 1999; Spiers and Maguire, 2006a; Bonnici et al., 2012) and self-referential (Johnson et al., 2002; Mitchell et al., 2005; Cabeza and St. Jacques, 2007) processing, and they indicate that the PFC can be involved in personal memory retrieval not only in experimental circumstances but also during real-life conditions.

We assume that these category-specific high gamma effects in association areas cannot be explained by articulation- or movement-related differences between conditions for the following reasons: First, these effects occurred outside the sensorimotor cortex. Second, no significant differences in sensorimotor-cortical gamma responses between the investigated conditions mirrored gamma effects in association areas in any subject. Third, there were no correlations of high gamma responses with the IU word count.

### Other cognitive functions

Beyond mnemonic functions, the differential effects in the IPC and in the PFC regions may also be related to other higher-order processes. Both areas have been implicated in intention perception (Fogassi et al., 2005) and intentional behavior (Thinnes-Elker et al., 2012). As the production of mnemonic contents in real-world conversations usually corresponds to the speaker's effective intentions, the differences in neuronal activity between the investigated conditions may also be related to the speaker's *intentions* to express memory- vs. non-memory-related content.

The category-specific neuronal effects in the present study could not be explained by different numbers of words between IU categories. Word number has been shown to be a reliable index of syntactic complexity in quantitative linguistic research (Szmrecsanyi, 2004), and an explanation of the observed category-specific responses by systematic differences in syntactic complexity is therefore unlikely. However, since oral speech comprises various levels of description involving articulation, word retrieval, short-term working memory, coordination with communication partners, and a multitude of other processes (Price, 2012), systematic coupling of such communication-related features with mnemonic content is an important topic for future research. Further investigation of psychological and linguistic differences between cognitive units may be of interest. For example, one may classify IUs based on the degree and valence of emotional content, or based on their syntactic properties.

To sum up, the present non-experimental, IU-based approach shows that human cognition can be studied in a real-life environment, and that IUs provide a handle to quantitatively explore such higher-order functions as naturalistic mnemonic processing in the human brain. Our behavioral and neuronal findings indicate that IUs can be used to decompose long conversations into small, self-contained units which (i) elicit robust speech-related activations in the articulatory areas and (ii) reflect differential IU contents in higher-order association regions. Since IUs in real-world speech production comprise a wealth of information about natural human cognition, future research in this direction can be expected to shed more light on the neuronal basis of brain functions which enable social discourse in real-world conditions.

### Outlook

As summarized by Auer (2010), many other ways exist to segment spoken language into basic meaningful elements, e.g., according to its prosodic and semantic characteristics. Since prosody has been proposed to reflect the boundaries of thoughts more directly than sound-based elements of speech (Chafe, 2012), and considering that spontaneously spoken language contains “many instances in which prosodic and syntactic units fail to coincide” (Chafe, pers. commun.), an interesting question for future neurolinguistic investigations will be to explore the neuronal differences between units obtained using alternative segmentation approaches, and to find out the borders of which unit types are most clearly reflected in brain activity. Application of hierarchical clustering algorithms (e.g., unsupervised learning) on ongoing neuronal activity recordings during spontaneous speech may be used to assess the success of linguistic segmentation. Beyond spatially localized effects at the level of single electrodes, large-scale dynamical network states may provide segmentation-relevant information.

Single-trial decoding of IU-related activity in the present study proved difficult, as IU classes could only be decoded from single ECoG trials in one subject (S1) with an accuracy of 61.6%, and significant decoding of IU subclasses was not possible in the remaining subjects. Future analyses with different decoding algorithms, different features, or based on signals from electrodes with a higher spatial resolution such as micro-ECoG (Gierthmuehlen et al., 2011; Bouchard et al., 2013) may result in a better decoding performance. If feasible, decoding the content of IUs from single trials of neuronal activity may further aid restoration of intended speech output in paralyzed patients with articulatory impairments (Pei et al., 2011; Derix et al., 2012; Pasley et al., 2012).

Apart from the taxonomy by Dritschel (1991) which we have made use of in the present study, other approaches exist to classify units of cognition by their content. For instance, one may distinguish between IUs describing events vs. states, whether a reference is made to a situation which is immediate or displaced, whether it is factual or fictional, and if the given IU conveys a belief, intention, or desire. Chafe (1994) proposes these and several other ways of classification. It may be interesting in future neurolinguistic investigations into non-experimental, spontaneous communication to elucidate the neuronal activation patterns peculiar to these IU classes.

A further question that merits attention with regard to mnemonic processing is how different levels of recency are reflected in neuronal activity during recollection. Bonnici et al. (2012) performed a comparison of neuronal activation patterns in fMRI while the subjects thought about recent vs. old memories, and these authors obtained topographically-specific results in the ventromedial PFC and in the hippocampus. Future ECoG studies of spontaneous speech may classify the subjects' recollections in a similar way or perhaps attempt temporally more fine-grained differentiation. Since German is a language with multiple past tenses which could provide indications of recency of the recollected situation, tense information may be useful to detect IUs with different temporal references.

A relevant further question which can be addressed with the present approach would be whether memory retrieval in real-world conversations differs when the subject is directly asked about a past event, compared to a situation in which mnemonic processing is triggered intrinsically. What is the impact of the conversation partner on how many and which memories are accessed and how? Do neuronal mechanisms of memory retrieval differ when subjects talk about a topic discussed in the directly preceding utterance, compared to a new utterance which relates to a new topic? Studying these and many related questions can be possible by analyzing natural, uninstructed conversations. With regard to psycholinguistic research, Neisser stated in 1978 that “the naturalistic study of memory is an idea whose time has come.” We assert that ECoG obtained during non-experimental communication is a rich source of information for cognitive studies in the neuroscientific domain.

Last but not least, the change in content of IUs over time may merit attention. As temporal sequences of IUs are intimately linked to the flow of thoughts in the course of spontaneous speech (Chafe, 1984, 2012), deciphering patterns in the temporal structure of IU production in larger speech epochs than investigated in the present study may be a way to address these cognitive dynamics. Linguistic approaches to information structure analysis (Heusinger, 1999) or psycholinguistic methods to identify more and less likely temporal patterns of cognitive unit precedence (Spiers and Maguire, 2008) may be useful to address this largely unexplored question in future neurolinguistic studies on real-world communication (Chafe, 2012).

### Conflict of interest statement

The authors declare that the research was conducted in the absence of any commercial or financial relationships that could be construed as a potential conflict of interest.
