# Supplementary material for: From speech to thought: the neuronal basis of cognitive units in non-experimental, real-life communication investigated using ECoG
Source: Front Hum Neurosci. 2014 Jun 13;8:383. doi: 10.3389/fnhum.2014.00383 (PMC4056309; doi:10.3389/fnhum.2014.00383)
Supplement: Supplementary file 1 [file DataSheet1.DOCX]

**Supplementary Table 1.** Examples of the different IU categories in our data. Grey font denotes parts of the clauses which do not belong to the IUs.

|  | **German** | **English translation** | **basic properties** |
| --- | --- | --- | --- |
| **Idea Units (IUs)** | (a) Das machen wir ja morgens und abends.  (b) …, dass sie dahin kommen kann. | (a) We do it in the morning and evening.  (b) … that she can come here. | A main clause (a) or a subordinate clause (b) which consists of a finite verb plus all its modifiers. |
| **Memory Units (MUs)** | (a) Der war richtig spannend.  (b) Ich habe viele Interessen. | (a) It was really interesting.  (b) I have many interests. | Explicit (a) or implicit (b) reference to the past. |
| **Non-Memory Units (nMUs)** | Das kannst du auch in den Papierkorb werfen. | You can throw it in the wastepaper basket too. | Reference to the current situation. |
| **Personal Memory Units (PMUs)** | Das machen wir ja morgens und abends. | We do it in the morning and evening. | Explicit reference to self. |
|  | Die haben es jetzt abends gemacht. | Now they did it in the evening. | Implicit reference to self, the speaker witnessed the referred state/ event. |
| **Non-Personal Memory Units nPMUs)** | Als sie am Mittwochabend nach hause gekommen sind… | When they came home on Wednesday evening… | No reference to self, the speaker did not witness the referred state/ event |
| **Metamemory Units** | wodran ich mich erinnere, … | what I remember… | Information about memory and the speaker's ability to access it. |
| **Autobiographical Memory Units (AMUs)** | Das machen wir ja morgens und abends. | We do it in the morning and evening. | Action with explicit reference to self. |
|  | Die haben es jetzt abends gemacht. | Now they did it in the evening. | Implicit reference to self, the speaker witnessed the referred state/ event. |
| **Autobiographical Facts** | Ich habe viele Interessen. | I have many interests. | Factual information about the speaker. |
| **Prospective Memory Units** | Ich mache jetzt heute und morgen noch das Gleiche. | I am doing today and tomorrow still the same [thing]. | Memory for future plans. |
| **Actions** | (a) Die war ja nicht mehr da.  (b) Da finden die Stimulierungen statt. | (a) She was no longer here.  (b) Then the stimulations take place. | Contain verbs which explicitly (a) or implicitly (b) refer to a past state/ event. |
| **Evaluations** | Der war richtig spannend. | It was really interesting. | Interpretation of a previous state/ event. |
| **Propositional Attitudes** | Ich habe gar nicht auf einen Anruf von ihm gerechnet. | I did not expect his call at all. | Explicitly in past tense (Dritschel, 1991), denotes an attitude, thought, doubt, belief, or intention. |
| **Reported Speech** | Hat sie gesagt, doch, macht sie natürlich. | She said she would certainly do it. | A clause containing reported speech. |
